# Method for extracting elemental sulfur in environmental water and its application to blue tide samples from Tokyo Bay, Japan

**DOI:** 10.1007/s44211-025-00717-9

**Published:** 2025-01-24

**Authors:** Shogo Sugahara, Hiroto Higa, Mitsuki Iwama, Yoshiyuki Nakamura, Tetsunori Inoue, Yukiko Senga, Michiko Egawa, Yasushi Seike

**Affiliations:** 1 Department of Chemistry, Graduate School of Natural Science and Technology, Shimane University, 1060 Nishikawatsu, Matsue, Shimane 690-8504 Japan; 2 Institute of Urban Innovation, Yokohama National University, Hodogaya, Yokohama, Kanagawa 240-8501 Japan; 3 Graduate School of Science and Engineering, Shimane University, 1060 Nishikawatsu, Matsue, Shimane 690-8504 Japan; 4 Sediments Management Association, 3-10-9 Irifune, Chuo-ku, Tokyo, 104-0042 Japan; 5 Marine Environment Control System Department, Port and Airport Research Institute, 3-1-1 Nagase, Yokosuka, Kanagawa 239-0826 Japan; 6 Estuary Research Center, Shimane University, 1060 Nishikawatsu, Matsue, Shimane 690-8504 Japan; 7 Department of Transdisciplinary Science and Engineering, Tokyo Institute of Technology, 4259 Nagatsuta-Cho, Midori-Ku, Yokohama, Kanagawa 226-8503 Japan; 8 College of Life and Environmental Sciences, Wenzhou University, Wenzhou, 325035 Zhejiang China; 9 Faculty of Sciences, Toho University, 2-2-1 Miyama, Funabashi, Chiba 274-8510 Japan

**Keywords:** Elemental sulfur, Environmental water, Spectrophotometry, Blue tide, Tokyo bay

## Abstract

A simple method for determining elemental sulfur in environmental water was developed and applied to seawater samples collected immediately after the occurrence of blue tides in Tokyo Bay. To investigate the concentration and extraction methods, artificial elemental sulfur was quantitatively produced by oxidizing a sulfide solution with an iodine solution, then used as a standard reagent in the experiments. To concentrate the elemental sulfur in the water sample, glass filter paper (GF/F) was used to filter and collect the elemental sulfur. The elemental sulfur was then extracted using *n*-hexane, the main component of petroleum ether; however, the recovery of elemental sulfur from the wet glass filter paper was low, and remained so even when the glass filter paper was dried. We, therefore, used a mixture of *n*-hexane and an acetone solvent, which is a hydrophilic organic solvent, for extraction and succeeded in recovering more than 90% of the elemental sulfur from the wet glass filter paper. Using this solvent mixture, we extracted and quantified elemental sulfur from seawater samples collected after the occurrence of blue tide, and detected 0.36–0.38 mgS L^–1^ of elemental sulfur in the near-surface layer. We also found that the elemental sulfur concentrations were higher in the surface layer than in the bottom layer. Therefore, we demonstrated that the quantification of elemental sulfur is important to better understand the blue tide phenomenon.

**Graphical abstract:**

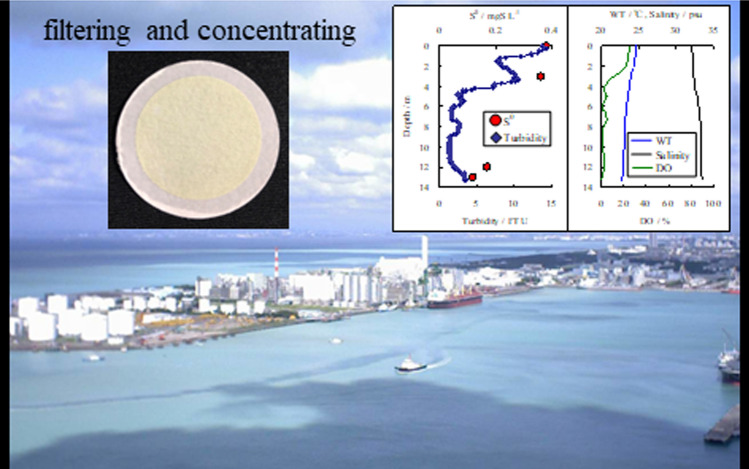

## Introduction

A blue tide is a phenomenon in which water containing hydrogen sulfide rises in shallow waters owing to wind and other physical factors. As a result, the hydrogen sulfide chemically oxidizes near the surface to form elemental sulfur, giving the water a bluish-white color. Although blue tides occur frequently in Tokyo Bay, Japan [1], few studies have quantified the formed elemental sulfur after blue tides. This is because it is difficult to predict the occurrence of blue tides, and because elemental sulfur produced by the oxidation of hydrogen sulfide diffuses and settles over time, making observation difficult. This is partly because of the lack of a simple method for determining elemental sulfur in environmental water.

Elemental sulfur has been detected in soils [2–5], crude oil [6], hydrocarbons [7], and pharmaceuticals [8]. As it is almost insoluble in water but soluble in organic solvents, chloroform [2], carbon disulfide [9], and petroleum ether [7] are used to extract elemental sulfur from samples. The concentration of elemental sulfur is then determined by ICP [2], HPLC [3, 4, 8], and titration methods [10]. There are also absorption spectrophotometric methods for determining elemental sulfur, such as adding chromium (II) chloride solution to the sample and reducing elemental sulfur to hydrogen sulfide for determination [11–13], or extracting elemental sulfur with an organic solvent, then adding sodium cyanide and ferric chloride and measuring the absorbance of iron thiocyanate (FeSCN^2+^) [7, 14]. However, the method of reducing elemental sulfur to hydrogen sulfide requires extensive equipment to gasify and collect the generated hydrogen sulfide, making it difficult to pretreat a large number of samples simultaneously. On the other hand, the iron thiocyanate method uses highly toxic KCN, and the stability of the complex (FeSCN^2+^) produced is relatively low [15]. However, iron thiocyanate method is advantageous for application to environmental water samples in that it is simpler than the method for analyzing hydrogen sulfide by reducing elemental sulfur in a series of operations. In addition, the equipment (spectrophotometer) is inexpensive compared to ICP or HPLC, and its maintenance and handling are easy, making absorbance spectrophotometry highly important even today, when instrumental analysis has been developed. Therefore, in this study, we established a series of methods for concentrating, extracting, and quantifying elemental sulfur from samples collected after blue tide occurrence based on the method of Bartlett and Skoog (1954) [7], with some modifications for analyzing water samples. The series of methods were then applied to seawater samples collected after a blue tide in Tokyo Bay on August 17, 2017, and the elemental sulfur content was successfully determined.

## Experiment

### Reagents and apparatus

To prepare the elemental sulfur standard solution (100 mgS L^–1^): 0.050 g of powdered sulfur (special grade, FUJIFILM Wako Pure Chemical Corporation) was dissolved in 500 mL of *n*-hexane (special grade, FUJIFILM Wako Pure Chemical Corporation); working standards were prepared by serially diluting with *n*-hexane before use (dynamic range: 0.05–20 mgS L^–1^). To prepare a 95% acetone solution: 950 mL of 99.5% acetone (special grade, FUJIFILM Wako Pure Chemical Corporation) was added to 50 mL of ion exchanged water (IEW) to make 1 L of the acetone solution. To prepare the potassium cyanide–95% acetone solution: 0.050 g of potassium cyanide (FUJIFILM Wako Pure Chemical Corporation, special grade) was dissolved in 500 mL of the 95% acetone solution. To prepare the ferric chloride–95% acetone solution: 0.50 g of ferric chloride hexahydrate (FUJIFILM Wako Pure Chemical Corporation, special grade) was dissolved in 500 mL of the 95% acetone solution; after 24 h, the solution was filtered using a glass filter paper (GF/F, Whatman), and the filtrate was used for the experiments. To prepare the solvent mixture for elemental sulfur extraction: 15 mL of *n*-hexane and 5 mL of the 95% acetone solution were mixed. To prepare the sulfide ion solution for producing artificial elemental sulfur: 1.0 g of sodium sulfide 9-hydrate was dissolved in 100 mL of deoxygenated IEW using N_2_ gas. To prepare the 0.05 M iodine solution: 20 g of potassium iodide (special grade, FUJIFILM Wako Pure Chemical Corporation) was dissolved in 100 mL of IEW; then, 12 g of iodine (special grade, FUJIFILM Wako Pure Chemical Corporation) was added and dissolved to the solution, and sufficient IEW was added to make the volume of the solution 1000 mL. To prepare the 0.1 M sodium thiosulfate solution: 25 g of sodium thiosulfate pentahydrate (special grade, FUJIFILM Wako Pure Chemical Corporation) was dissolved in 1000 mL of IEW. To prepare the starch solution: 1 g of soluble starch (special grade, FUJIFILM Wako Pure Chemical Corporation) was dissolved in heated IEW; after cooling to room temperature, the IEW was added to a final volume of 100 mL. To prepare the 0.1 M magnesium chloride solution: 20.0 g of magnesium chloride hexahydrate (special grade, FUJIFILM Wako Pure Chemical Corporation) was dissolved in 1000 mL of IEW. A UV–visible spectrophotometer (Shimadzu Corporation, UV1800) was used to measure absorbance. The shaker used in this study was EYELA MMS-300 (TOKYO RIKAKIKAI CO., LTD.).

#### Making the artificial elemental sulfur (1.0 mgS)

Artificial elemental sulfur was produced quantitatively by standardizing sulfide ion solutions via the redox reaction of hydrogen sulfide and iodine solution (Eq. 1) to produce elemental sulfur:

1 $$H_{2} S + I_{2} \to \, S^{0} + 2H^{ + } + 2I^{ - }$$

First, 1.0 g of sodium sulfide 9-hydrate was dissolved in 100 mL of deoxygenated IEW. Then, 20 mL of the 0.05 M iodine solution was placed in an Erlenmeyer flask, to which 10 mL of the sulfide ion solution and 1.0 mL of concentrated hydrochloric acid (special grade, FUJIFILM Wako Pure Chemical Corporation) were added; the mixture was placed in the dark for 5 min, then titrated with the 0.1 M sodium thiosulfate solution, using the 1% starch solution as the indicator. Denoting the titration value as *a* mL and the blank test volume as *b* mL, the liquid volume (*X* mL) of the sulfide ion solution containing 1.0 mg of elemental sulfur was calculated using the following equation:

2 $$X\left( {{\text{mL}}} \right) = \frac{6.24}{{\left( {b - a} \right) \times f}}$$

*a*: dropping volume of 0.1 M sodium thiosulfate solution into 20 mL of 0.05 M iodine solution + 10 mL of sulfide ion solution, *b*: Dropping volume of 0.1 M sodium thiosulfate solution into 20 mL of 0.05 M iodine solution (blank test), *f*: Factor of 0.1 M sodium thiosulfate solution.

Next, *X* mL (= 1.0 mgS) of the sulfide ion solution, as determined by Eq. 2, was accurately pipetted and gently added to the bottom of a test tube containing 3 mL of the 0.05 M iodine solution, to which 0.1 mL of concentrated hydrochloric acid was added; the test tube was then placed in the dark for 5 min. Then, 3 mL of 0.1 M sodium thiosulfate was added to remove excess iodine. The artificial elemental sulfur produced was captured on a GF/F filter by suction filtration. Elemental sulfur is a hydrophobic colloid that undergoes peptization and passes through the paper when washed with water. When a small amount of electrolyte solution is added to a hydrophobic colloid, such as elemental sulfur, the colloidal particles aggregate to form large particles and precipitate (coagulation). Therefore, a 0.1 M magnesium chloride solution was used for washing the elemental sulfur, resulting in all remaining elemental sulfur in the test tubes being transferred to the GF/F filter.

#### Standard procedure for determining elemental sulfur

GF/F filters supplemented with elemental sulfur from water samples were placed in 25-mL test tubes, to which 20 mL of mixed solvent was added for elemental sulfur extraction, following which the lids were reinforced with plastic tape to prevent opening. Elemental sulfur was then extracted by shaking the test tubes at room temperature (about 20 °C) for 24 h. The shaking speed was set to approximately 70 rpm so that the gas phase in the test tubes moved significantly from side to side. Next, 5 mL of the supernatant solution was transferred to each test tube and 15 mL of the potassium cyanide–95% acetone solution and 20 mL of the ferric chloride 95% acetone solution were added. The absorbance at 465 nm was then measured using 5-cm cell.

## Results and discussion

### Solvents used for extraction of elemental sulfur

**Fig. 1:**
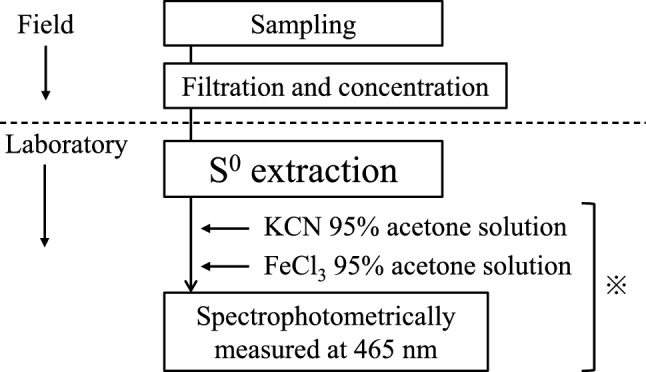
Flow of elemental sulfur from sampling to measurement. The * in the figure based on the method of Bartlett and Skoog [7]

While the elemental sulfur to be analyzed in this study was from environmental water, the concentrations of elemental sulfur in environmental waters can be extremely low. In addition, the determination of elemental sulfur content can be affected by positive errors arising from the presence of coexisting substances such as hydrogen sulfide. Therefore, we decided to concentrate the elemental sulfur and separate it from coexisting substances by suction filtrating the sample water using GF/F filter paper (pore size 0.7 µm), which has the smallest pore size among the glass filter papers tested for this study. To do so, the first step was to extract elemental sulfur from the GF/F filter supplemented with elemental sulfur (Fig. 1).

First, the elemental sulfur content used in the experiment was examined. When a certain amount of commercially available elemental sulfur standard reagent was weighed and suspended in IEW or MgCl_2_ solution, the elemental sulfur became a film on the water surface and could not be suspended. Even when suction filtration was performed using a large amount of rinse with IEW or MgCl_2_ solution, the elemental sulfur stuck to the edges of the filter, and it was not possible to transfer all the elemental sulfur collected onto the filter paper. This is thought to be due to the fine particle size of the elemental sulfur standard reagent. On the other hand, the elemental sulfur produced by the redox reaction of sulfide ion solution and iodine solution was obtained in suspension in the solution from the beginning, and all the produced artificial elemental sulfur could be transferred to the filter paper. Therefore, in this study, this artificial elemental sulfur was used as the standard sample.

Next, experiments were conducted to determine the solvents to be used for extracting elemental sulfur. In this method, it is necessary to use the supernatant solution after extraction and to consider sufficient prewashing of the volumetric pipette when transferring the sample to the test tube. Therefore, the volume of the extraction solution was set at 20 mL. A GF/F filter with 1.0 mgS of artificial elemental sulfur trapped was placed in a test tube while still wet with water and 20 mL of *n*-hexane was added, then allowed to stand for 48 h. Then, 5 mL of the supernatant solution was transferred to another test tube, to which 15 mL of a 95% potassium cyanide solution and 20 mL of a 95% ferric chloride solution in acetone were added to determine the elemental sulfur. This resulted in a mean recovery rate of 61% (Table 1). Shaking the test tube for 48 h increased the average recovery to 71%, but 100% recovery was not achieved. To attempt increasing the recovery, we dried GF/F filters with 1.0 mgS of elemental sulfur at 110 °C for 2 h or at 50 °C for 24 h, then placed them in 20 mL of *n*-hexane and subjected them to shaking for 48 h; however, this yielded low recoveries of only 17% and 13%, respectively. This suggests that elemental sulfur may have transitioned when the filter paper was heated to remove moisture. Therefore, it was necessary to extract elemental sulfur from wet filter paper to achieve efficient extraction.

**Table 1:** Extraction of elemental sulfur using 20 mL of *n*-hexane

Filter condition	Extraction condition	Added	Found	Recovery	RSD
		mgS	mgS	%	%
Wet^a^	Stand for 48 h	1.0	0.64 0.58 0.55 0.66	61	8.1
Wet^a^	Shake for 48 h	1.0	0.72 0.69 0.71 0.70	74	1.9
Drying at 110 °C for 2 h	Shake for 48 h	1.0	0.18 0.17 0.18 0.16	17	7.4
Drying at 50 °C for 24 h	Shake for 48 h	1.0	0.12 0.14 0.14 0.11	13	12

^a^Wet means that the filter paper contains water after filtration

Therefore, we mixed *n-hexane* with a hydrophilic organic solvent and extracted elemental sulfur from wet filter paper. Acetone, which is used in the preparation of coloring reagents, was used as the hydrophilic organic solvent. Four solvent mixtures were prepared by changing the ratios of *n*-hexane to 95% acetone in the solutions, and 20 mL of each solution was added and shaken for 48 h. As a result, we obtained excellent recoveries of more than 90% when the solvent compositions were 15 mL *n*-hexane + 5 mL 95% acetone and 10 mL *n*-hexane + 10 mL 95% acetone (Table 2). We selected the 15 mL *n*-hexane + 5 mL 95% acetone solvent mixture for extracting elemental sulfur as it afforded the highest recovery. It was also found that at least GF/F glass filter paper (pore size 0.7 µm) should be used for filtering and concentrating elemental sulfur.

**Table 2:** Extraction of elemental sulfur using various solvent mixtures

Extraction solvent	Added	Found	Recovery	RSD
*n*-Hexane/mL + 95% Acetone/mL	mgS	mgS	%	%
15 + 5	1.0	0.92 0.86 0.97 0.94	92	5.2
10 + 10	1.0	0.84 0.92 0.92 0.94	90	4.6
5 + 15	1.0	0.65 0.62 0.74 0.72	68	8.2
0 + 20	1.0	0.57 0.57 0.64 0.56	58	6.3

### Shaking time for the extraction of elemental sulfur

**Fig. 2:**
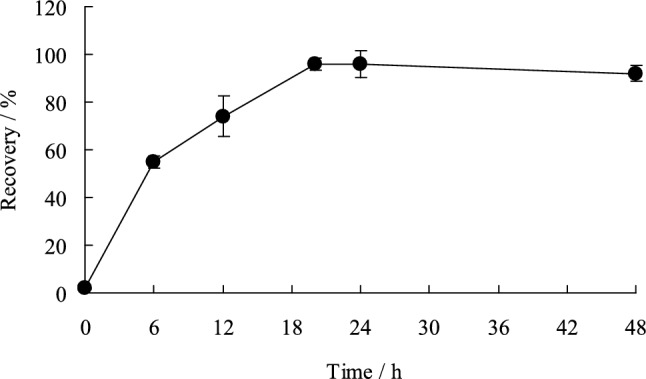
Shaking time for the extraction of elemental sulfur. Black circle denote average values (*n* = 4), error bars indicate standard deviations. Test conditions: S^0^ concentration = 1.0 mgS, shaken using a shaker (about 20 °C, 70 rpm)

GF/F filter containing 1.0 mgS of elemental sulfur was placed in a test tube, to which 20 mL of the chosen solvent (15 mL *n*-hexane + 5 mL 95% acetone) was added, following which the test tube was shaken (about 20 °C, 70 rpm). After 20 h, the recovery of elemental sulfur was almost 100% (Fig. 2). The optimal shaking time for the extraction of elemental sulfur was determined to be 24 h.

### Filter paper for trapping elemental sulfur

Various types of glass filter paper were also tested for trapping elemental sulfur. Briefly, suction filtration was used to trap 1.0 mg of artificial elemental sulfur each on three glass filter papers: GF/F (pore size 0.7 μm), GS-25 (pore size 1.0 μm; Advantec), and GF/C (pore size 1.2 μm; Whatman). As shown in Table 3, all three filter papers exhibited recoveries of more than 90% when the elemental sulfur was extracted using 20 mL of the 15 mL *n*-hexane + 5 mL 95% acetone solvent. Some elemental sulfur, formed by oxidizing hydrogen sulfide in environmental water, has a particle size smaller than the pore size of the GF/F glass filter paper [16]. However, the recovery rate of GF/F glass filter paper was as high as 95%. Therefore, GF/F glass filter paper was used as the filter paper to trap and concentrate elemental sulfur in environmental water.

**Table 3:** Examination of filter paper used to capture elemental sulfur

Filter (pore size)	Added	Found	Recovery	RSD
	mg	mg	%	%
GF/F (0.7 µm)	1.0	1.03 0.92 0.93 0.91	95	5.7
GS25 (1.0 µm)	1.0	0.89 0.92 0.97 0.83	90	6.6
GF/C (1.2 µm)	1.0	0.86 0.90 0.90 0.95	90	4.0

### Preservation of filter paper that captured elemental sulfur

A preservation method for the elemental sulfur trapped in GF/F filters was tested. GF/F filters containing 1.0 mgS of elemental sulfur were wrapped in aluminum foil and stored under freezing (–20 °C), refrigerated (4 °C), and room temperature (about 20 °C) conditions for 7 days. Then, they were placed in test tubes, to which 20 mL of the 15 mL *n*-hexane + 5 mL 95% acetone solvent was added; the test tube was then shaken for 24 h to extract the elemental sulfur. As shown in Table 4, excellent recoveries of more than 90% were obtained for the samples that were refrigerated and frozen. Therefore, it was concluded that after capturing elemental sulfur in the field, the filter paper can be kept stable for at least 7 days if wrapped in aluminum foil and refrigerated or frozen.

**Table 4:** Storage of filter paper

Condition	Added	Found	Recovery	RSD
	mg	mg	%	%
– 20 °C	1.0	0.94 0.88 0.92 1.00	94	5.2
4 °C	1.0	0.92 0.90 0.86 0.95	91	4.3
20 °C	1.0	0.81 0.90 0.89 0.72	83	9.0

### Application to environmental samples

**Fig. 3:**
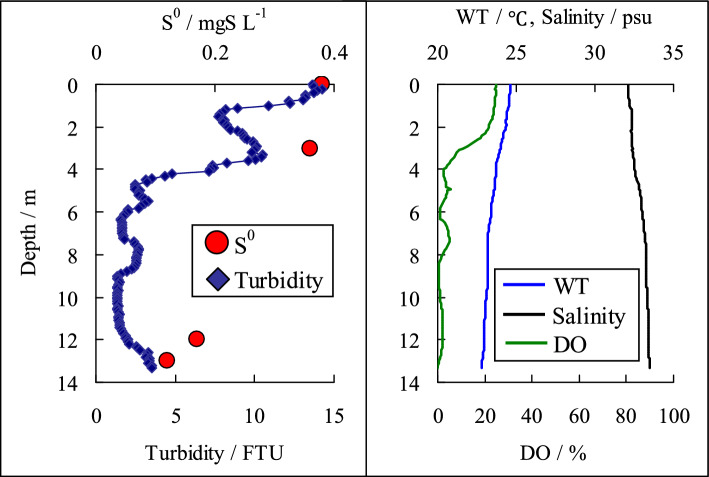
Vertical distribution of elemental sulfur (S.^0^), turbidity, water temperature (WT), salinity and dissolved oxygen (DO) during blue tide (sampling date: August 17, 2017)

A blue tide occurred in Tokyo Bay on August 17, 2017. The survey was conducted by a ship offshore Funabashi in Tokyo Bay, and sample water was collected vertically from the ship. After collecting the seawater, 250 mL of it was immediately filtered on a GF/F filter to trap the elemental sulfur in the sample. The GF/F filters were wrapped in aluminum foil and kept cold until back to the laboratory and were then frozen until analysis. Turbidity, temperature, salinity, and dissolved oxygen were measured vertically using a water quality sensor (AAQ-176 RINKO, JFE Advantech). Using our developed method, the elemental sulfur in the seawater sampled during a blue tide occurrence was determined: 0.36–0.38 mgS/L of elemental sulfur was detected in the samples collected from depths of 0 and 3 m in the sea, where turbidity values were high (Fig. 3). In contrast, the elemental sulfur concentrations from the samples collected at depths of 12 and 13 m (near the seafloor) ranged from 0.12 to 0.17 mgS L^−1^. This showed that elemental sulfur concentrations were higher near the water surface than in the bottom layer. A blue tide is a phenomenon in which water containing dissolved sulfides rises in shallow water owing to wind and other physical factors. As a result, the dissolved sulfides react with the dissolved oxygen near the surface to form elemental sulfur; furthermore, consequently, the dissolved oxygen rapidly decreases. The results obtained from this survey are good indicators confirming the blue tide phenomenon. Blue tides cause anoxia in the surrounding sea (Fig. 3), which significantly impacts organisms; however, there are many unknowns regarding blue tides. We expect that this method will be useful for observing blue tides and developing measures to control them.

## Conclusions

In this study, a method was developed for the concentration, extraction, and colorimetric determination of elemental sulfur in environmental waters. Artificial elemental sulfur, prepared by the reaction of sulfide ions and iodine solutions, was used in the elemental sulfur concentration and extraction experiments. Given that the filter paper was wetted with water after trapping elemental sulfur, *n*-hexane could not be used to extract elemental sulfur. By mixing *n*-hexane with acetone, almost all the elemental sulfur was extracted from the wet filter paper. Furthermore, it was determined that the filter paper with trapped elemental sulfur was stable for at least 1 week when frozen.

Therefore, the procedure from sampling to the analysis of elemental sulfur in environmental water is as follows:

1. A certain amount of water containing elemental sulfur was filtered in the field, and elemental sulfur was trapped on the GF/F filter paper. The filter paper, containing the elemental sulfur, was frozen until analysis.

2. A filter paper containing elemental sulfur was placed in a test tube, and a solvent mixture was added for elemental sulfur extraction (*n*-hexane 15 mL and acetone 5 mL) and shaken for 24 h at room temperature.

3. The supernatant was transferred to another tube, two chromogenic reagents were added sequentially, and the absorbance was measured at 465 nm.

This method was applied to blue tide samples from Tokyo Bay, and 0.36–0.38 mgS/L of elemental sulfur was detected in the surface layer. In the future, this method can be useful not only for observing blue tides but also for elucidating the behavior of sulfur species.

## Data Availability

The data sets generated during and/or analyzed during the current study are available from the corresponding author on reasonable request.
